# Carbon Sequestration and Fertility after Centennial Time Scale Incorporation of Charcoal into Soil

**DOI:** 10.1371/journal.pone.0091114

**Published:** 2014-03-10

**Authors:** Irene Criscuoli, Giorgio Alberti, Silvia Baronti, Filippo Favilli, Cristina Martinez, Costanza Calzolari, Emanuela Pusceddu, Cornelia Rumpel, Roberto Viola, Franco Miglietta

**Affiliations:** 1 FoxLab, Forest & Wood Science, Fondazione E.Mach, San Michele all’Adige (Trento), Italy; 2 MountFor Project Centre, European Forest Institute, San Michele all’Adige (Trento), Italy; 3 Department of Agricultural and Environmental Sciences, University of Udine, Udine, Italy; 4 Institute of Biometeorology, National Research Council, Firenze, Italy; 5 European Academy of Bolzano, Bolzano, Italy; 6 Institute of Research for Hydrogeological Protection, National Research Council, Sesto Fiorentino (FI), Italy; 7 Biogéochimie et écologie des milieux continentaux, CNRS-INRA-ENS-Paris 6, Thiverval-Grignon, France; 8 Centre for Research and Innovation, Fondazione E.Mach, San Michele all’Adige (Trento), Italy; DOE Pacific Northwest National Laboratory, United States of America

## Abstract

The addition of pyrogenic carbon (C) in the soil is considered a potential strategy to achieve direct C sequestration and potential reduction of non-CO_2_ greenhouse gas emissions. In this paper, we investigated the long term effects of charcoal addition on C sequestration and soil physico-chemical properties by studying a series of abandoned charcoal hearths in the Eastern Alps of Italy established in the XIX century. This natural setting can be seen as an analogue of a deliberate experiment with replications. Carbon sequestration was assessed indirectly by comparing the amount of pyrogenic C present in the hearths (23.3±4.7 kg C m^−2^) with the estimated amount of charcoal that was left on the soil after the carbonization (29.3±5.1 kg C m^−2^). After taking into account uncertainty associated with parameters’ estimation, we were able to conclude that 80±21% of the C originally added to the soil via charcoal can still be found there and that charcoal has an overall Mean Residence Time of 650±139 years, thus supporting the view that charcoal incorporation is an effective way to sequester atmospheric CO_2_. We also observed an overall change in the physical properties (hydrophobicity and bulk density) of charcoal hearth soils and an accumulation of nutrients compared to the adjacent soil without charcoal. We caution, however, that our site-specific results should not be generalized without further study.

## Introduction

Thermo-chemical conversion of organic material under limited oxygen supply, within a certain range of temperatures (200–1200°C), transforms biomass into bio-oil and syngas, which may be used as an energy source, and produces a carbonaceous co-product (i.e. biomass-derived Pyrogenic-C or charcoal or biochar [1]) which has been proposed as a tool to mitigate climate change and improve soil fertility [2]. A recent study [3] quantified the theoretical carbon (C) sequestration potential of biochar following its incorporation in agricultural soils at a maximum rate of 50 Mg C ha^−1^ to a depth of 0.15 m as 1.8 Gt CO_2_-C_equivalent_ per year. This estimate corresponds to 12% of current global anthropogenic C emissions and includes: a) direct C sequestration, associated with the burial of recalcitrant organic C forms [4]; (b) potential reduction of N_2_O and CH_4_ emissions from soils associated with biochar application [5]; and (c) CO_2_ emissions avoided due to fossil fuel substitution by the energy released by biomass during pyrolysis and gasification. Moreover, several studies have shown that the addition of biochar to both poor and fertile agricultural soils may have beneficial effects on plant yields, thus amplifying its environmental benefit. These effects are associated with improvements in soil physical [6] and chemical properties [7], microbiological activity [8], temperature increase due to changes in surface albedo [9], hormesis (i.e. favorable biological responses to low exposures; [10]), as well as combinations of several of these different drivers [8].

However, while short-term studies have confirmed the potential of biochar to increase C storage and to improve soil physico-chemical properties in the short-term, the long-term effects of incorporating large amounts of pyrogenic C into the soil remain rather elusive. The actual ability of biochar to act as a C sink into the soil remains controversial due to uncertainties related to its long term stability [11]. Thousand-year old charcoal residues identified in archeological sites and areas interested by wildfires have been considered a demonstration of its long term stability in soils [12] [13] even though some studies have outlined the fact that the amount of Pyrogenic Carbon measured in soils is much lower than what would have been expected according to other paleontological and archeological artifacts [14] or the frequency and intensity of fires [15]. Rapid transformations of charcoal in soils by abiotic and biotic oxidation can occur [16] and its stability varies according to the initial feedstock, the charring conditions, and the environmental characteristics of the burial site [17]. Very ancient charcoal deposits, such as Terra Preta de Indio in Amazonia [18] and Bronze Age human settlements of Terramare in the Po Valley in northern Italy [19], are still rich in C and are still fertile substrates [20] [21].

The present study aimed to explore the centennial time scale stability of pyrogenic C incorporated as charcoal in soil. To do this, we used Alpine areas where charcoal, produced in traditional charcoal piles, was added to the soil more than one century ago and was not mixed with other organic sources. Moreover, we were able to assess the effect on physio-chemical soil properties after char addition to soil.

## Materials and Methods

### Ethics Statement

Collection of soil samples was authorized by the Stelvio National Park and Trentino Forest Service.

### Site Description and Soil Sampling

The study site is located in Val di Pejo (Trentino, Northern Italy; 46°20′16.18″ N, 10°36′07.02″ E) at an elevation ranging from 2120 to 2170 m a.s.l. The mean annual temperature at the site is 3.5°C and the mean annual precipitation is 903 mm [22]. The lowest precipitations are registered in January, while the highest are distributed between April and November.

Starting from the 16^th^ century the area was subject to intensive wood resource exploitation for larch charcoal production which was subsequently used in the local iron industry. Production ceased in 1858 when a severe fire event destroyed the major iron foundry in the valley [13] [23]. Charcoal production was based on large forest clear-cuts, wood chopping and downhill transportation to artificial flat terraces (charcoal hearths) with an elliptical shape. Some of these hearths are still identifiable today as terraces where wood piles were prepared and subsequently covered by soil and tree branches [24]. The relatively large size and elliptical shape of the hearths suggest that more than one pile of wood was carbonized at a particular time as it was already well known that only circular piles could ensure a uniform and high quality charcoal production [25]. Wood carbonization required between four and ten days according to the dimension of the wood pile. After a two-day cooling down period, the charcoal was finally transported to the foundry.

**Figure 1:**
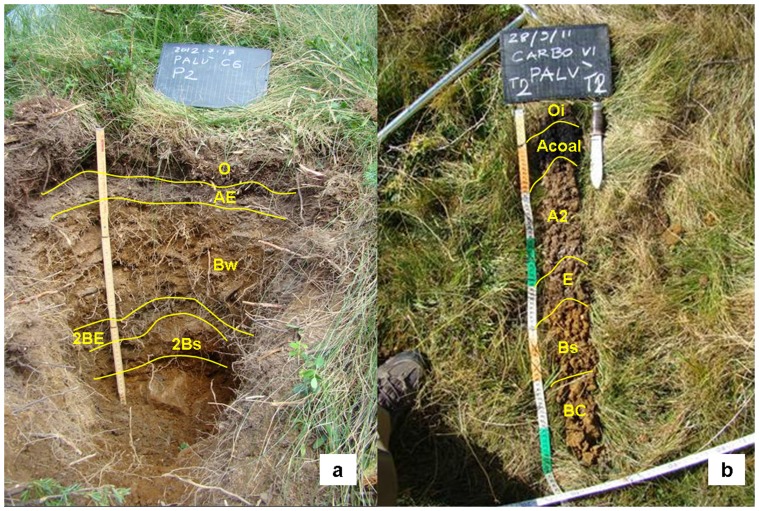
Soil profile at the control site (Panel a) and the charcoal hearth (Panel b). The letters indicate different pedologic horizons. In the charcoal hearth the dark anthropogenic layer (Acoal; 0–10 cm) can be easily identified.

Three hearths and three adjacent control areas with southerly aspects and unaffected by significant geo-morphological dynamics (erosion or sedimentation) or recent anthropogenic disturbances were selected. These are flat (2% slope) and have an average surface area (*s*) of 94 m^2^. Soils within the control areas are shallow to moderately deep (35–70 cm), sandy-loam brown acid soils and with restricted areas of podzols (Lithic Dystrudept and Entic Haplorthod according to USDA, 2010 [26]) with an approximate 25% slope. Soils in the charcoal hearths show a truncated profile, with a shallow surface organic horizon approximately 2 cm deep covering a thicker (19.3±2.8 cm) black anthropogenic layer. This horizon contains a large amount of charcoal fragments and fine particles left after carbonization which have been subsequently incorporated and well mixed with the pre-existing soil in response to bioturbation [27] and freeze-thaw processes [28] (Figure 1).

Both control soils and charcoal hearths are, nowadays, covered by the same herbaceous vegetation dominated by *Nardus stricta L*. while the surrounding forest is dominated by *Larix decidua* L. and *Picea abies* L.

The exact date of charcoal production was assessed using a dendro-anthracological approach. This method relies on cross-dating tree ring widths in charcoal fragments with known tree chronologies and has been used previously by [29], who showed that the oldest and youngest tree rings identified in charcoal fragments at our study area were dated 1530 and 1858 respectively. This last date corresponds to the year in which a wildfire event down in the valley caused the destruction of industrial plants thus determining the interruption of the local iron industry and charcoal production in the area [13].

### Soil Sampling and Chemical Analysis

The anthropogenic layer within the charcoal hearths was sampled using a manual soil corer at five different sampling points in each hearth. Similarly, the soil at approximately the same depth was sampled at five points in each control area. Soil samples were dried for 72 hours at 35°C and sieved to 2 mm. In the case of the charcoal hearths, the fraction of soil >2 mm was further separated into two subsamples, one including charcoal fragments and one including plant debris, roots and stones. All further analysis was completed on the five sampling points separately.

Soil pH was measured in a soil/water solution (1∶2.5 ratio). Soil C and N contents were determined by dry combustion using a CHN elemental analyzer (© Perkin Elmer 2400 series II CHNS/O elemental analyzer). Total Ca, K, Mg, Na, P, and available Ca, K, Mg, P concentrations were determined for subsamples oven-dried at 105°C for 24 h according to the EPA method 3052 [30] and the filtered solutions were analyzed using an ICP-OES spectrophotometer (Varian Inc., Vista MPX). A further set of subsamples was used to assess NO_3_^–^N according to the method proposed by [31] and NH_4_^+^-N according to the method proposed by [32]. Hydrophobicity was measured following the method of [33]. Such term defines the affinity for soils to water controlling infiltration or wetting. It can be caused by coating of long-chained hydrophobic organic molecules on individual soil particles in response to the decay of organic matter but also to the diversity of soil micro-organisms. Increased hydrophobicity is normally observed after wildfires that leaves charcoal fragments at the soil surface.

C and N content, and total and available Ca, K, Mg, Na, P concentrations of >2 mm charcoal individual fragments were determined using the same methodology described above. Micrographs of those charcoal fragments were made using a Scanning Electron Microscope, XL 20 FEI SEM, with CRYO-GATAN ALTO 2100 technology on samples dried under vacuum, following standard procedures [34].

To enable a comparison between old and fresh charcoal, fragments of larch wood were carbonized in a muffle furnace at 400°, 500°, 600°, 860°C. The time needed to complete the carbonization corresponds to the time needed for the sample to stabilize its weight loss. C and N contents were determined on samples using the methodology described above.

### Pyrogenic C Determination

The relative contribution of charcoal-C (C_CHAR_ : C_TOT_) to total soil carbon (C_TOT_) was estimated using a mass balance method [35] based on the δ^13^C values of charcoal fragments excavated from the anthropogenic soil layer (δ^ 13^C_CHAR_), the mean δ^ 13^C of the entire layer (δ^13^C_TOT_) and the δ^ 13^C of the SOM contained in the adjacent control soils (δ^13^C_SOM_) (Table 1):
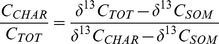
(1)


**Table 1:** Parameters, coefficients and variables used to distinguish and quantify the different carbon pools in charcoal soil layer (means ± standard error, n = 3).

Parameter/coefficient/variable	Definition	Unit	Value	Source(s)
δ^13^C_TOT_	Isotopic signature of the bulk anthropogenic soil layer in hearths	‰	−24.72±0.14	Measured with IRMS
δ^ 13^C_CHAR_	Isotopic signature of charcoal fragments extracted from theanthropogenic soil layer in hearths	‰	−24.53±0.01	Measured with IRMS
δ^ 13^C_SOM_	Isotopic signature of soil organic matter of control soils	‰	−26.28±0.30	Measured with IRMS
δ^ 13^C-CO_2HEARTHS_	Isotopic signature of respired CO_2_ from incubated hearth soils	‰	−25.22±1.13	Measured with CRDS andKeeling plot
δ^ 13^C-CO_2CONTROL_	Isotopic signature of respired CO_2_ from incubated control soils	‰	−24.81±0.31	Measured with CRDS andKeeling plot
C_TOT_	Carbon content of anthropogenic soil layer in hearths	kg C m^−2^	26.2±5.3	Measured and calculated
C_CHAR_	Pyrogenic carbon content of anthropogenic soil layer in hearths	kg C m^−2^	23.3±4.7	Calculated with mass balance
C_SOM_	Soil organic matter content of anthropogenic soil layer in hearths	kg C m^−2^	2.9±0.6	Calculated with mass balance

Stable C isotope ratio (δ^13^C) measurements were made on the fine fraction (<2 mm) of representative subsamples of control and hearth soils using an Isotope Ratio Mass Spectrometer (© Thermo Fischer Scientific, Delta V Plus) following total combustion in an elemental analyser (© EA Flash 1112 ThermoFinnigan).

The δ^13^C signature of respired CO_2_ from incubated charcoal hearth and control soils was measured using the Picarro G2131-*i* δ^ 13^C High-precision Isotopic CO_2_ Cavity Ring Down Spectrometer (CRDS) and Keeling plot method [36]. Representative subsamples (∼250 cm^3^; n = 3) were incubated in Erlenmeyer flasks at 40°C for 15 minutes. Air was continuously circulated from the flask to a pump and then back into the flask at a rate of 0.8–1.0 l min^−1^. The CRDS was connected to the pump inlet tube and the air sub-sampled at 0.015 l min^−1^ for measurements of CO_2_ concentration and δ^13^C. Sampling frequency was 0.5 Hz. To determine the δ^13^C signature of the respired CO_2_, the Keeling method was applied [36]. The intercept of the linear regression with the y-axis represents the isotopic signature of the source of the flux. Regression coefficients were calculated on averaged data at each 50 ppm interval of CO_2_ concentration, starting from 450 to 800 ppm to establish a steady mixing within the flask. Finally, mean and standard deviation values of δ^13^C were computed for both charcoal hearth and control soils (n = 3). We assumed that any difference in the δ^13^C of SOM in control and charcoal hearths would also be reflected by a difference in the δ^13^C of the respired CO_2_.

### Net C Sequestration

Ancillary information that is required to estimate the net C sequestration in hearths’ soils was gathered from different sources:

the amount of wood that was harvested and used for the carbonization process (*W_s_*) was estimated on the basis of forest stand volume at three sub-alpine larch forests of varying ages. One forest was located on the slopes above the hearths of Val di Pejo, one in the nearby Val di Rabbi and one in Val Comasine. All forests had the same altitude (2000 m) and the latter two sites are known to be old-growth forests where 600 year old larch trees can still be found. Stand volume (m^3^ ha^−1^) was estimated using Light Detection And Ranging (LiDAR) measurements processed according to [37] and converted into biomass using an average wood basal density of 860 kg m^−3^. For each forest, maximum wood stock was calculated using a fixed number of pixels;
forest surface area that supplied each hearth during charcoal production (*h*), was determined by geo-referencing the charcoal hearths and drawing a 26 ha polygon on a digital orthophoto (Terraitaly™; © Compagnia Generale Ripreseaeree S.p.A. – Parma). *h* was calculated according to the following two criteria: i) the wood collection area should be located above each charcoal hearth up to the tree line; ii) the lateral boundaries of the area corresponded to the mean distance between two charcoal hearths (∼100 m). Finally, the total area was divided by the total number of hearths identified in the area (n = 7).
carbonization efficiency, i.e. the ratio between produced charcoal and used wood (*q*), was assumed to be equal to 20% according to [38];
the fraction of charcoal that was left on the soil surface at the end of each carbonization cycle after char was removed by charcoal makers (*w*), was experimentally estimated using a modern analogue. A charcoal hearth that is currently in use was identified at short distance from the Val di Pejo, where expert charcoal makers repeat traditional charcoal production mainly for didactical purposes. They also recorded, year by year, the exact amount of wood used and of charcoal produced. The amount of pyrogenic C left on hearth soil was quantified using the loss-on-ignition (LOI) method [39] using four replicates randomly selected within the hearth area. *w* was determined as the ratio between the sum of C contained in the charcoal that was made over the last ten years and the corresponding amount of C that was found in the soil. The uncertainty was estimated as the standard error of the mean;
C content of freshly produced larch wood charcoal (*r_0_*) was also assessed experimentally. Known amounts of larch wood taken from wood disc collected in the proximity of Val di Peio charcoal hearths were pyrolized at different temperatures in a muffle furnace. The C concentration was measured on each charcoal sample using a CHN elemental analyzer. Measured C content data were fitted to production temperature using a second order polynomial relationship and C content at a reference temperature of 450°C was assumed to be an analog of the charcoal originally produced [40].

### Uncertainty and Sensitivity Analysis

All data in the text and in the tables are reported as mean ± standard error (n = 3) if not differently indicated.

Gaussian error propagation technique (GEP) was used in error analysis to analytically determine the error or uncertainty produced by multiple and interacting measurements or variables. For this, the uncertainty associated to each measurement was calculated as standard error (se) of the mean (se = standard deviation/root square of the number of samples) and the classical error propagation theory and equations were used [41]. Error sensitivity analysis was also made by constructing an error budget [42], thus enabling further understanding of the error structure, i.e. the relative contribution of the errors associated with each parameter to the overall error estimate. Such sensitivity indices provide in this way a measure of the percentage rate of change in an output variable produced per unit percentage change in its input variable, an information that may be used critically to identify where error reduction of estimates may lead to lower uncertainty.

## Results and Discussion

### Soil Bulk Density and Hydrophobicity

The anthropogenic layer of the charcoal hearth soils has a lower bulk density than control soils (0.60±0.08 vs. 0.87±0.12 Mg m^−3^; p<0.01). Soil bulk density is an important indicator of physical soil quality, being linked to air capacity, resistance to root growth and capacity of storing and transmitting water [43]. Such a decrease in bulk density is associated with a 97.3% ±1.6 decrease in hydrophobicity. This is in line with the water infiltration data from charcoal production sites in Ghana [44], but in contrast with short term observations following biochar applications to soil [45] [46] that showed small but consistent increases in soil hydrophobicity which is largely controlled by the surface chemistry of fresh biochar particles [47]. We speculate that a prolonged residence time of charcoal caused substantial leaching or degradation of hydrophobic compounds [48], a shift in soil texture ([49], [44]), and a microbially-driven creation of functional groups [50]. Decreased hydrophobicity is known to increase water availability for plants and is also important for nutrient cycling, as it favors water infiltration into the soil and reduces runoff, thus preventing lateral nutients losses.

### Nutrients and Carbon

Nutrient content (total and plant available fractions) is higher in the hearths than in the control soils (Table 2; Table 3). In particular, the total P-stock is 107% larger in hearths than in the control (95±6 vs. 46±3 g m^−2^, p = 0.003), while the plant available P is 24% higher (1.2±0.04 g m^−2^ vs. 0.9±0.3 g m^−2^). The higher P content is not surprising as charcoal contains at least 20% of P originally contained in the wood (Table 2). If we assume that charcoal made in the middle of the XIX century had a P content comparable to that of modern charcoal (3.0±0.05 g P kg^−1^; Table 3), we may estimate that carbonization events led to the addition of 117±21 g P m^−2^ (Table 2). In the absence of grass mowing, a virtually closed P-cycle can be hypothesized for these soils as P-leaching does not usually occur if the overall concentrations are low so that P can be considered “virtually immobile” [51] [52]. Nevertheless, large herbivores are known to be net P-exporters in alpine grasslands as they may preferentially graze in P-enriched areas and then release P as dung elsewhere [53]. This export largely depends on the grazing pressure, but it is unlikely to exceed the maximum value of 0.07 g P m^−2^ y^−1^ that was assessed experimentally in the Swiss Alps [53]. When scaled to the time that charcoal was added to the soil the amount of P exported would not have exceeded 11.5 g m^−2^. Atmospheric deposition may have also contributed to the P balance as a result of long-range desert dust transport and as a consequence of atmospheric pollution, including biomass combustion [54]. Although the latter is known to be variable in time and space, this input is not large in the Eastern Alps, with less than 0.01 g P m^−2^ y^−1^ [55]. When measured today, the total amount of P contained in the anthropogenic layer of the hearth's soil is only 19% less than what was initially added during the carbonization events (95±6 vs. 117±21 g m^−2^; Table 2). This highlights the fact that charcoal production was indeed a long-lasting source of P in an otherwise P-limited environment. P-fertilization persisted on a centennial time-scale and it is also interesting to observe that both organic and inorganic (extractable) fractions of P that were added to the soil are now mostly contained in the non-pyrogenic fraction of the SOM, as the P contained in old charcoal fragments is only 12% of that of modern laboratory-produced larch charcoal (Table 3).

**Table 2:** Total carbon and nutrient stocks in the control soils and charcoal hearths and the estimated amount added by carbonization calculated according to Eq. [2].

Element	control soils	charcoal hearths	p-value	Input by carbonization
C (kg m^−2^)	8.1±0.3	26.2±5.3	0.03	29±5
P (g m^−2^)	45.8±3.1	95±7	0.003	117±21
K (g m^−2^)	231±34	285±70	0.53	112±20
Ca (g m^−2^)	136±23	368±80	0.10	229±41
Mg (g m^−2^)	127±88	62±5	0.51	59±10
N (g m^−2^)	582±84	500±29	0.42	80±15

Mean ± standard error (n = 3). Results of the comparison between control and charcoal hearth (p-value) are also reported.

**Table 3:** Total and available nutrient concentrations (mg kg^−1^ dry matter ± standard error; n = 3) measured in control soils, charcoal hearths, old and new charcoal fragments and larch wood.

	Control soils		Charcoal hearths		Old charcoal fragments	Fresh charcoal fragments	Larch wood
Element	Total concentration	Available	Total concentration	Available	Total concentration	Total concentration	Total concentration
Ca^2+^	993±135	278±35	3300±185	1006±158	3438±275	5920±70	5334±53
K^+^	1603±116	147±30	2463±69	279±120	885±66	2899±39	1415±77
Mg^2+^	2739±73	80±12	2378±384	245±4	1215±76	1533±32	1158±12
Na^+^	297±50	34±1	86±8	33±26	216±1	207±4	190±4
P	321±7	7±2	921±206	12±4	346±81	3005±53	2716±46
N-NO_3_^−^	1.96±0.74	–	2.34±0.89	–	–	–	–
N-NH_4_^+^	3.93±1.00	–	3.73±1.80	–	–	–	–

**Figure 2:**
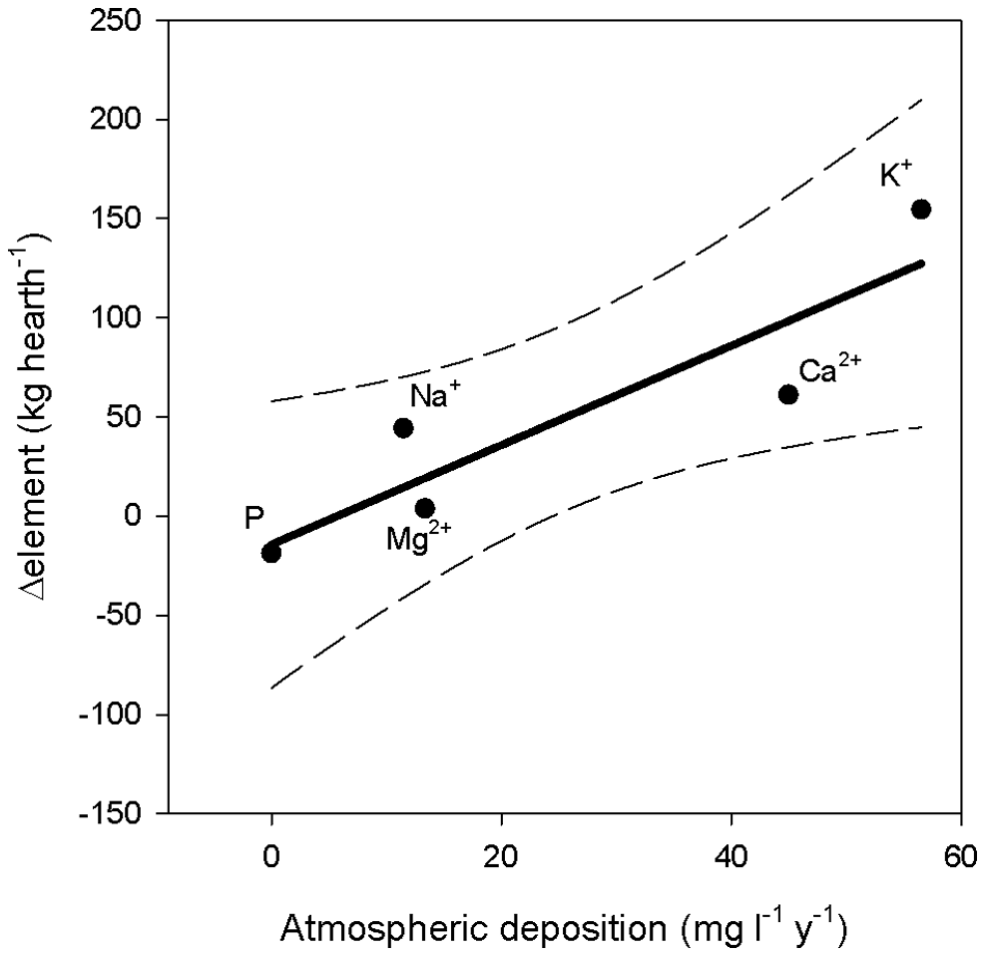
Correlation between average annual atmospheric deposition of P, K^+^, Ca^2+^, Mg^2+^, Na^2+^ (mg l^−1^ y^−1^) and the difference between the input of the same elements due to charcoal application in 1858 and the amount found today in hearth’s soils (Δelement, kg hearths^−1^) (y = 2.50×–14.31, R^2^ = 0.82, p = 0.035). Dashed lines represent 95% confidence interval.

Other nutrients, such as potassium (K) and calcium (Ca) are also more abundant in the charcoal hearth soils even though there are no significant differences with control soils due to the large spatial variability (Table 2). Furthermore K and Ca are 155% and 61% higher than what was added to the soil with charcoal (Table 2). This excess may be attributed to an higher retention of atmospheric K and Ca depositions which have been previously reported to be relevant in the Alpine region [56] and can be related to the higher cation exchange capacity (CEC) of charcoal [57]. A strong correlation between current atmospheric deposition rates and element excesses found in the hearth soil seems to confirm such hypotheses (Figure 2).

Total N content is not significantly different between hearth and control soils (p = 0.42; Table 2) and no significant difference was found in the concentrations of mineral N (NO_3_^−^: p = 0.31; NH_4_^+^: p = 0.92; Table 3).

Total C content of the anthropogenic layer at the charcoal hearths is three times higher than that of the adjacent control soils (p = 0.03; Table 1). The amount of C that is now contained in the anthropogenic soil layer (C_TOT_, kg C m^−2^) is the sum of pyrogenic (C_CHAR_, kg C m^−2^) and non-pyrogenic components (C_SOM_, kg C m^−2^) as carbonates are absent due to the low pH (4.2±0.3 and 4.6±0.3 in charcoal hearths and control areas, respectively). Charcoal fragments larger than 2 mm represent approximately 4.1±1.7% (by weight) of the entire mass of the anthropogenic layer within the deeper soil horizons where no charcoal debris can be identified (Figure 1).

Any meaningful evaluation of net C sequestration achieved in charcoal hearths firstly requires a precise separation of C fractions contained in the charcoal (C_CHAR_) and in the rest of the soil, followed by a accurate estimation of the C input at the time of the carbonization event.

**Figure 3:**
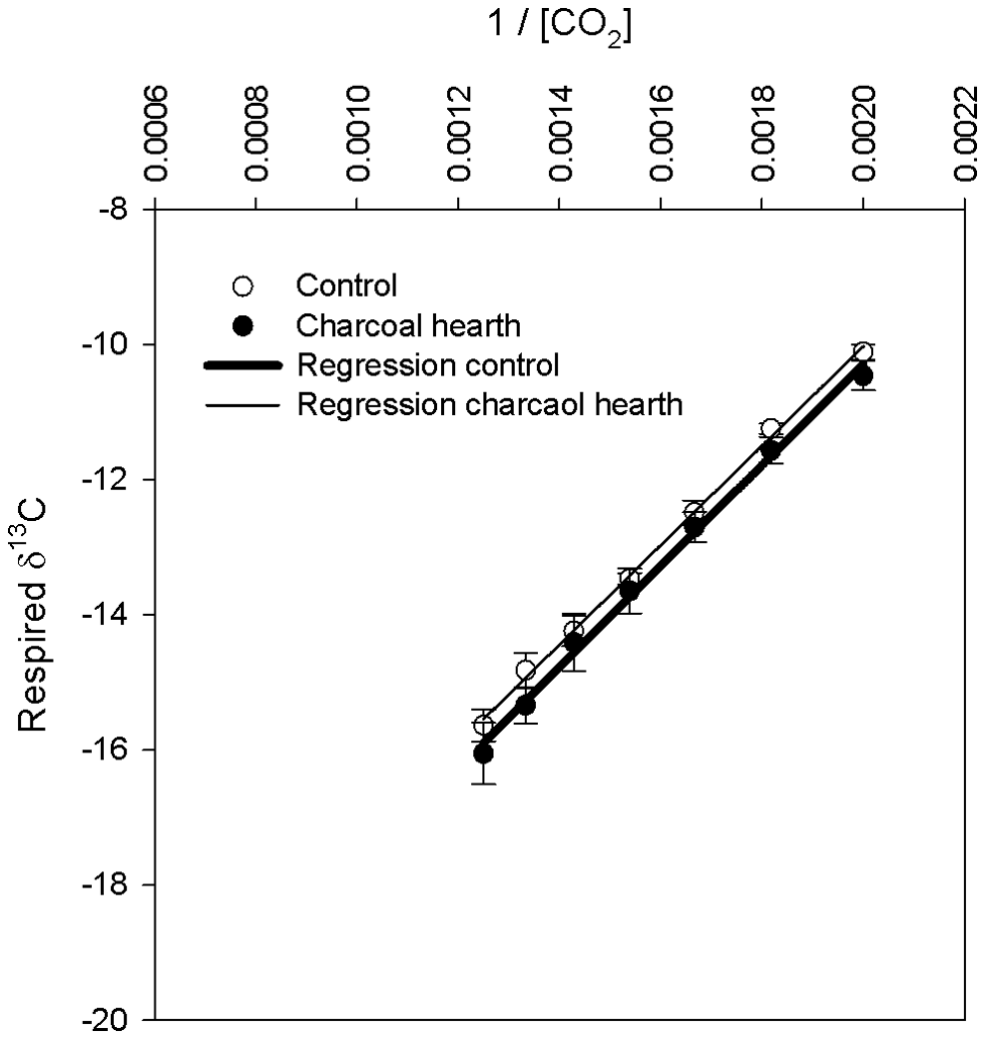
Keeling plots measured by CRDS showing the δ^13^C of respired CO_2_ fluxes versus the reciprocal of CO_2_ concentration for control and charcoal hearth incubated soils (δ^13^C_CONTROL_ = 7353*[CO_2_]^−1^−24.8, R^2^ = 0.99; δ^13^C_CHARCOAL HEARTH_ = 7467*[CO_2_]^−1^−25.2, R^2^ = 0.99). Horizontal and vertical bars indicate standard deviations (n = 3).

**Figure 4:**
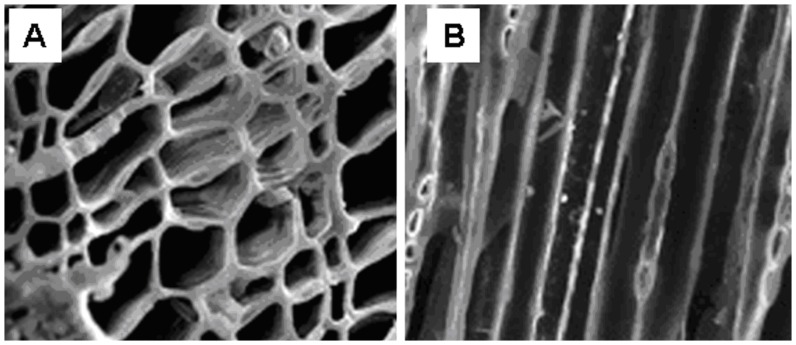
SEM micrographs showing the inner morphology of charcoal fragments and the absence of any microbes or plant debris. a) is a radial section b) a longitudinal section.

Assessing the exact ratio between C_CHAR_ and C_TOT_ using sable C isotopes is problematic because:

Equation 1 assumes a net difference in the isotopic signature of old charcoal and modern SOM. This is supported by the observation that wood formed before 1900 is on average ∼1‰ less negative than that formed after 1950 [58] due to the recent rapid rise in δ^13^C-depleted atmospheric CO_2_ concentrations as a result of fossil fuel burning [59]. In addition, it has been reported that branch or stem wood of C_3_ plants is generally enriched by 1–3‰ compared to leaves [60]. Despite the fact that limited ^13^C-depletion may occur during wood carbonization at temperatures above 300°C [61], it could be expected that the charcoal fragments found in the hearth’s soils are significantly enriched in the heavier C isotope compared to the more recent SOM pools that are mainly derived from the decomposition of litter formed more recently;
Equation 1 assumes that δ^13^C_SOM_ in the hearth’s soils is equal to δ^13^C_SOM_ in the control soils. Such equivalence cannot be assumed *a priori*, but CRDS-based δ^13^C flux measurements demonstrated that the signature of respired CO_2_ was not significantly different between control and charcoal hearth incubated soil (p = 0.75; Figure 3 and Table 1). Similarly, it may be assumed that the δ^13^C of the less recalcitrant (non-pyrogenic) SOM fractions is the same in both hearth and control soils. Such an equivalence is further confirmed by the linearity observed by plotting the reciprocal of the C content of charcoal fragments, hearth and control soils versus their respective stable C isotopic ratios (slope = −11.3; intercept = −24.3; r^2^ = 0.98; n = 9; p<0.0001). The fact that the data points fall within close proximity to a straight line indicates that the two C pools (pyrogenic and non-pyrogenic C) are distinguished in the hearth’s soils;
The δ^13^C of the charcoal fragments (δ^13^C_CHAR_ in Equation 1) could be affected by the presence of organic debris or micro-organisms in charcoal pores. The analysis of charcoal fragments with a Scanning Electron Microscope showed that, despite being exposed in soil for over 150 years, no organic or inorganic debris were present in the inner portion of the fragments ([34]; Figure 4). A recent study [62] showed that, in spite of the large empty space in charcoal pores, these were only sparsely populated by microorganisms three years after application to soil. This was attributed to the adsorption of inorganic and organic compounds which may cause blockage of the charcoal pores and thus prevent microorganisms penetrating the inner portion of fragments. Our data support this observation, showing that charcoal, also in the long term, does not provide a habitat for microbes and, accordingly, the δ^13^C of the charcoal fragments is unaffected.

Based on the above assumptions and considerations, the fraction of pyrogenic C (C_CHAR_) contained in the overall soil C (C_TOT_) was finally estimated using Equation 1 to be equal to 89±9%, corresponding to an absolute amount of 23.3±4.7 kg of pyrogenic C m^−2^ (Table 1).

### Carbon Sequestration

Once the amount of pyrogenic C is known, a reliable estimation of the C sequestration achieved in the hearth soil over centennial timescale requires a proper estimation of the initial C input (C_IN_, kg C m^−2^). This can be obtained according to equation:
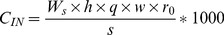
(2)


**Figure 5:**
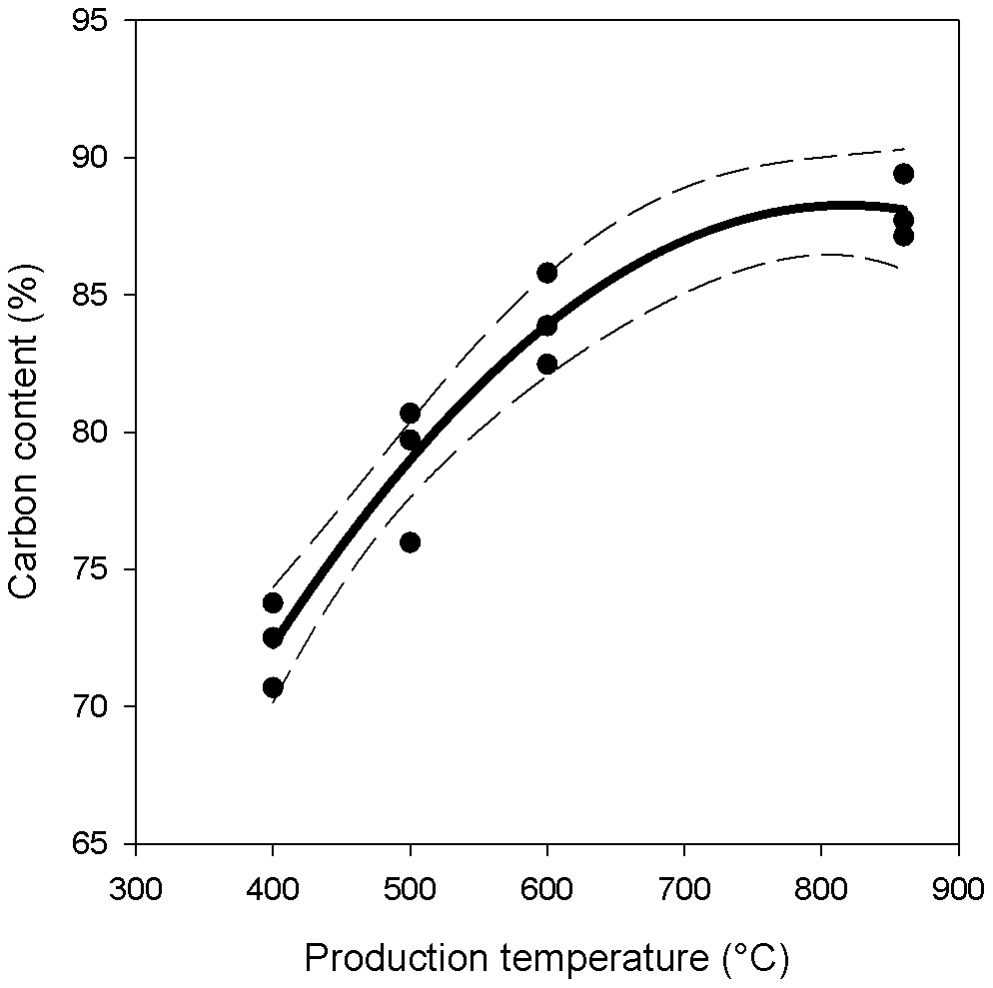
Carbon content of charcoal produced from larch wood at different temperatures. Wood samples were collected in close proximity to the hearths. Charcoal was produced in a muffle furnace at 400°, 500° 600° and 860°C. Dashed lines represent 95% confidence interval. (Y = 26.9+0.15 X-9.2 10^−5^ X^2^; r^2^ = 0.94; p<0.0001).

Solving Equation 2 is problematic mainly because of the uncertainty associated with the determination of parameters *W_s_*, *w* and *r_0_*:

the total amount of wood biomass (*W_s_*) that was used for carbonization cannot be directly estimated but requires the use of a proxy. The assumption can be made that the forest standing biomass one and half centuries ago was comparable to what is currently found in our study area (288±41 t ha^−1^; Table 4). This assumption is partly confirmed by the fact that LIDAR-based standing volumes of two additional old-growth forest sites (>200 years) are comparable to the wood stock of the study area. The fact that tree volume is independent of stand age is not surprising and is confirmed by the usually reported plateau of forest wood stocks over centennial times scales [63] in alpine larch forests [64];
the fraction of charcoal left on the soil after carbonization can only be estimated indirectly as there a no reliable sources reporting such a value. We assumed that an experimental assessment using modern charcoal hearth is the most reliable proxy of ancient charcoal hearths. The value obtained in this way (w = 0.02±0.002) was not far from judgment of two expert charcoal makers who unanimously said that no more than 2% charcoal fragments are normally left over the soil at the end of each carbonization cycle;
the C content of new charcoal (*r_0_*) is known to vary substantially with production temperature (r^2^ = 0.94, p<0.0001; Figure 5). Here again there are not historical data on temperature during charcoal production but FAO reports that the average temperature in traditional carbonization wood piles is around 450°C [40]. Using this value, *r_0_* was estimated to be equal to 0.76±0.004.

**Table 4:** Description of larch forests considered as analogues of the larch forest harvested for charcoal production in Val di Pejo (mean ± standard error).

Forest site	Coordinates	Elevation (m a.s.l.)	Forest age	Number of plots	Wood stock (W, t ha^−1^)
Val di Pejo	46°20′16.18″ N, 10°36′07.02″ E	2152	150	51	288±41
Val di Rabbi	46°26′39.45″ N, 10°45′59.56″ E	1864	500	20	213±27
Val Comasine	46°20′02.00″ N, 10°39′58.78″ E	2119	650	40	258±25

Data were determined using LiDAR measurements [37].

Based on the above assumptions and considerations, C_IN_ could be calculated to be equal to 29.3±5.1 kg C m^−2^ (Table 5) and the overall pyrogenic C lost was then quantified as:
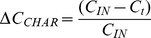
(3)where *C_t_* is the actual pyrogenic C in the soil and *t* is time since the last char production at the charcoal hearth (153 years). Thus, the fraction of pyrogenic C lost since the time of carbonization was equal to 0.20±0.28 of C_IN_ (Table 5). This value is given by the sum of the direct charcoal degradation by biotic and abiotic factors and the lateral transport due to surface runoff and erosion occurring during and after char production, before a new soil layer covered the anthropogenic layer [65]. The presence of small amounts of minute charcoal fragments predominantly down-slope of the hearths seems to confirm the occurrence of such lateral flows.

**Table 5:** Parameters, coefficients and variables used to estimate charcoal stability in soil (mean ± standard error; n = 3).

Parameter/coefficient/variable	Definition	Unit	Value	Source(s)
q	Carbonization efficiency	–	0.20	[38]
w	Fraction of charcoal left on the ground at theend of carbonization	–	0.02±0.017	Measured with LOI
r_0_	Carbon content of larch wood charcoal produced at 450°C	g g^−1^	0.76±0.040	Measured with CHN and extrapolation
r_155_	Carbon content of charcoal fragmentsfound in the hearth’s soil	g g^−1^	0.60±0.032	Measured with CHN
W_s_	Larch wood stock of the forest in Val di Pejo	t ha^−1^	288±41	Measured (LiDAR) and calculated to convertfrom m^3^ ha^−1^ to t ha^−1^
h	Forest area for wood collection for charcoalproduction per hearth	ha hearth^−1^	3.7	Measured on ortophotos
s	Surface area of charcoal hearths	m^2^	94	Measured
C_IN_	Pyrogenic carbon left on the ground at the time ofthe carbonization	Kg C m^−2^	29.3±5.1	Calculated using Eq. [2]
–	Fraction of pyrogenic carbon lost since the time ofthe carbonization calculated with Eq. [2]	–	0.20±0.28	Calculated
–	Fraction of pyrogenic carbon lost since the time of thecarbonization calculated with *r_0_* and *r_155_*	–	0.21±0.04	Calculated
k_CHAR_	Annual pyrogenic carbon decay rate in hearths	years^−1^	0.0015±0.0003	Calculated
MRT	Mean residence time of charcoal in the soil	years	650±139	Calculated

Recent studies have used changes of the C-content of individual charcoal fragments buried in the soil as a proxy of the fraction of carbon lost from charcoal over long time scales [66]. Such method is certainly questionable, as it cannot distinguish between actual C-losses due to oxidation and C-dilution effects associated to the deposition of inorganic salts and minerals on those fragments. Nevertheless, it is noteworthy, here, to highlight that the C content of individual charcoal fragments found in the charcoal hearth’s soils (*r_155_* = 0.60±0.03 gC g^−1^) is consistently lower than the C content of modern larch charcoal made in the laboratory (*r_0_* = 0.76±0.04 gC g^−1^). The fact that the relative difference between *r_0_* and *r_155_* (0.21) almost exactly matches our estimate of the fraction of C lost on centennial time scale (Equations 2 and 3) is likely coincidental but suggests that more detailed investigations on the oxidation of ancient charcoal fragments ([67]) and on mineral deposition on old charcoal fragments are needed.

The decay rate (*k*, years^−1^) can be finally calculated as:
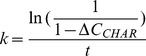
(4)and is equal to 0.0015±0.0003. Such a value is less than half of the value estimated in a recent meta-analysis of charcoal decomposition [68] and corresponds to a Mean Residence Time (MRT) of 650±139 years (Table 5).

Error sensitivity analysis performed on the results of Equation 2 and 3 illustrates the relative contribution of errors to the overall uncertainty of the result (Table 6). The data show that the largest contribution to the overall uncertainty is in the error associated to the anthropogenic soil layer depth. Such large error suggests that variance among the three replicated hearths may not be simply random but reflects, instead, some systematic effects possibly associated to differences in the amount of wood that was carbonized in each hearth. This is indeed a critical aspect, possibly requiring a re-analysis of the simplifying assumption of an even distribution of carbonization intensity (amount of wood carbonized) among the replicated hearths. Such re-analysis would in fact enable a substantial reduction of the uncertainty, while not affecting the means. Other important sources of error are related to the estimation of the C-content of the anthropogenic layers (C_tot_) and in the estimated amount of wood that was used for the carbonization process (W_s_). Error reduction would have been possible, in the first case, by increasing the number of replicates and more unlikely by increasing samplings in each replicate. Nevertheless, a relative large uncertainty would remain associated to the second case, as W_s_ was inferred on the basis of large simplification and that by no means new information could be retrieved to finally reduce its error.

**Table 6:** Uncertainty and sensitivity analysis results for the setimation of equation 3 parameters.

Variable	Variable uncertainty(standard error)	Relative contribution to the overalluncertainty in ΔC_CHAR_ in Eq.3
W_s_	41	16%
w	0.002	11%
r_0_	0.004	1%
Soil organic C content	0.03	18%
Soil bulk density	71	14%
Anthropogenic soil layer depth	0.04	26%
δ^13^C_TOT_	0.14	11%
δ^ 13^C_CHAR_	0.31	3%
δ^ 13^C_SOM_	0.01	1%

## Conclusions

This study presents two key messages. First, it provides novel insights into the long-term decomposition of pyrogenic C in the soil, by demonstrating that charcoal addition to soil is indeed a way to obtain substantial C-sequestration. Despite some inevitable uncertainty, we have shown that 23.3±4.7 kg C m^−2^ of pyrogenic C are still present in the soil after an addition of 29.3±5.1 kg C m^−2^ that was made in the middle of the XIX century. Carbon sequestration was estimated as 80±21% of the original added C. Secondly, our investigation provides substantial evidence that the avaliability of macro- and some micro-nutrients is higher in charcoal hearth soils over centennial timescales. This supports the common observation that the addition of various forms of pyrogenic C (biochar) increases soil fertility and plant yield, even in the long-term. The persistence of enhanced nutrient avaliability over centennial timescales is likely associated with mechanisms favoring their accumulation and improving soil water relations. Overall, this strongly supports the idea that the addition of biochar to soil is indeed a feasible, effective, and sustainable strategy to both sequester atmospheric C and to enhance crop yields.

However, we call for some caution on excessive generalization of our results: the hearths within the Val di Pejo are located in a mountainous area, at high elevations, and are exposed to peculiar climatic conditions, that are certainly different to the vast majority of areas where croplands are concentrated. Even if soil freeze-thaw cycles associated with large seasonal temperature fluctuations are likely to favour decomposition and C oxidation, it is not certain if and how the C decomposition rates that we observed are greater or smaller than in other climates. Additional caution is required since that the charcoal added to the studied hearths came from a single feedstock (larch wood) and was produced in traditional charcoal production systems. The fate and the effects of other feedstocks and of other production processes such as slow and fast pyrolysis, pyrogasification, and hydrothermal conversion may indeed create totally different biochar types, possibly behaving in different ways in the soil.

Charcoal hearths in Val di Pejo are certainly a unique resource for investigating the long term effect of pyrogenic C addition to the soil. The number of replications which are available, the long residence time of the charcoal in the soil, the accuracy of charcoal dating, the reliability of ancillary information on the sequence of events that were associated to charcoal production and its sudden cessation, contribute to the scientific value of these sites. A number of questions, not considered in the present study, could be addressed in the near future, such as, for instance, the effect of long-term charcoal burial on (i) its physio-chemical properties; (ii) non-CO_2_ greenhouse gas fluxes (CH_4_ and N_2_O); (iii) plant productivity; and (iv) shifts and changes in soil biodiversity.
